# Mitochondrial glycerol 3-phosphate facilitates bumblebee pre-flight thermogenesis

**DOI:** 10.1038/s41598-017-13454-5

**Published:** 2017-10-12

**Authors:** Stewart W. C. Masson, Christopher P. Hedges, Jules B. L. Devaux, Crystal S. James, Anthony J. R. Hickey

**Affiliations:** 1 School of Biological Sciences, University of Auckland, 3a Symonds St, Auckland, 1010 New Zealand; 2 Institute of Sport, Exercise and Active Living, Victoria University, Melbourne, VIC Australia

## Abstract

Bumblebees *(Bombus terrestris)* fly at low ambient temperatures where other insects cannot, and to do so they must pre-warm their flight muscles. While some have proposed mechanisms, none fully explain how pre-flight thermogenesis occurs. Here, we present a novel hypothesis based on the less studied mitochondrial glycerol 3-phosphate dehydrogenase pathway (mGPDH). Using calorimetry, and high resolution respirometry coupled with fluorimetry, we report substrate oxidation by mGPDH in permeabilised flight muscles operates, *in vitro*, at a high flux, even in the absence of ADP. This may be facilitated by an endogenous, mGPDH-mediated uncoupling of mitochondria. This uncoupling increases ETS activity, which results in increased heat release. Furthermore, passive regulation of this mechanism is achieved via dampened temperature sensitivity of mGPDH relative to other respiratory pathways, and subsequent consumption of its substrate, glycerol 3-phosphate (G3P), at low temperatures. Mitochondrial GPDH may therefore facilitate pre-flight thermogenesis through poor mitochondrial coupling. We calculate this can occur at a sufficient rate to warm flight muscles until shivering commences, and until flight muscle function is adequate for bumblebees to fly in the cold.

## Introduction

Bumblebees, such as the burrowing bumblebee (*Bombus terrestris*), undergo pre-flight thermogenesis to facilitate flight at low temperatures^1–4^. While this is well established, the mechanisms mediating pre-flight thermogenesis are not fully understood. There are two main hypotheses, futile cycling and shivering. In futile cycling, phosphofructokinase and fructose 1,6-bisphosphatase simultaneously convert fructose 6-phopshate to fructose 1,6- bisphosphate and vice versa. This stimulates heat production via increased ATP hydrolysis, and the resulting ADP increasing ETS activity^5,6^. While this has been an example in biochemistry texts, estimates based on maximal enzymatic capacities suggest that this process likely generates less than 7% of the pre-warming heat required to enable flight^7^. Shivering also releases significant heat given that the actin-myosin interaction is thermodynamically inefficient^8^, and also increasing ADP concentrations will stimulate heat production from the ETS. However, flight muscle contraction is arrested below 15 °C, and insufficient for shivering below 25 °C^9^. Indeed, shivering is the current explanation for hymenopteran flight muscle heating, and given that there is minimal lactate dehydrogenase (LDH) expression^10^ or phosphogen transfer system^11,12^, all flight muscle ATP is generated through oxidative phosphorylation (OXPHOS). It remains unknown whether bumblebee flight muscle mitochondria can adequately support ATP-dependent heating processes such as shivering, or can respire to release sufficient heat to warm muscles independent of OXPHOS.

In bumblebees, mitochondria occupy approximately 40% of flight muscle volume, and account for some of the highest mass specific respiration rates measured^13^. Moreover, flight muscle mitochondria have high activities of mGPDH, which is also highly expressed in BAT and has been suggested to have a thermogenic function in mammals^14–17^. To sustain glycolytic flux, the glycerol 3-phosphate (G3P) pathway effectively oxidises cytosolic NADH. G3P is produced by cytosolic GPDH through the reduction of dihydroxyacetone phosphate using NADH formed during glycolysis^16^. However, mGPDH then converts NADH (Δ*G*°′ − 220 kJ mole^−1^) to FADH_2_ (Δ*G*°′ − 181.6 kJ mol^−1^), imparting a 17.5% loss in reducing power. This energy loss may be resolved as heat. Moreover, the coupling of FADH_2_ to OXPHOS results in ~40% less ATP per oxygen reduced, and equates to approximately 288 kJ mol^−1^ loss of energy relative to NADH, assuming Δ*G*′ of ATP = −72 kJ mol^−1^ ^18,19^.

To address mitochondrial contributions to heat release we measured respiration and real-time ATP synthesis rates from permeabilised flight muscles, across temperatures representative of hypothermic and euthermic states. We hypothesised that 1) the low bioenergetic efficiency of G3P-supported respiration would be reflected in measures of mitochondrial function (i.e. respiration and ATP synthesis rates) and that this would result in substantial heat release. Furthermore, we hypothesised that 2) this respiratory pathway would maintain function at low temperatures associated with pre-flight warming and finally that 3) changes in metabolite abundance would support a role for mGPDH in *B. terrestris* thermogenesis.

## Results and Discussion

**Figure 1:**
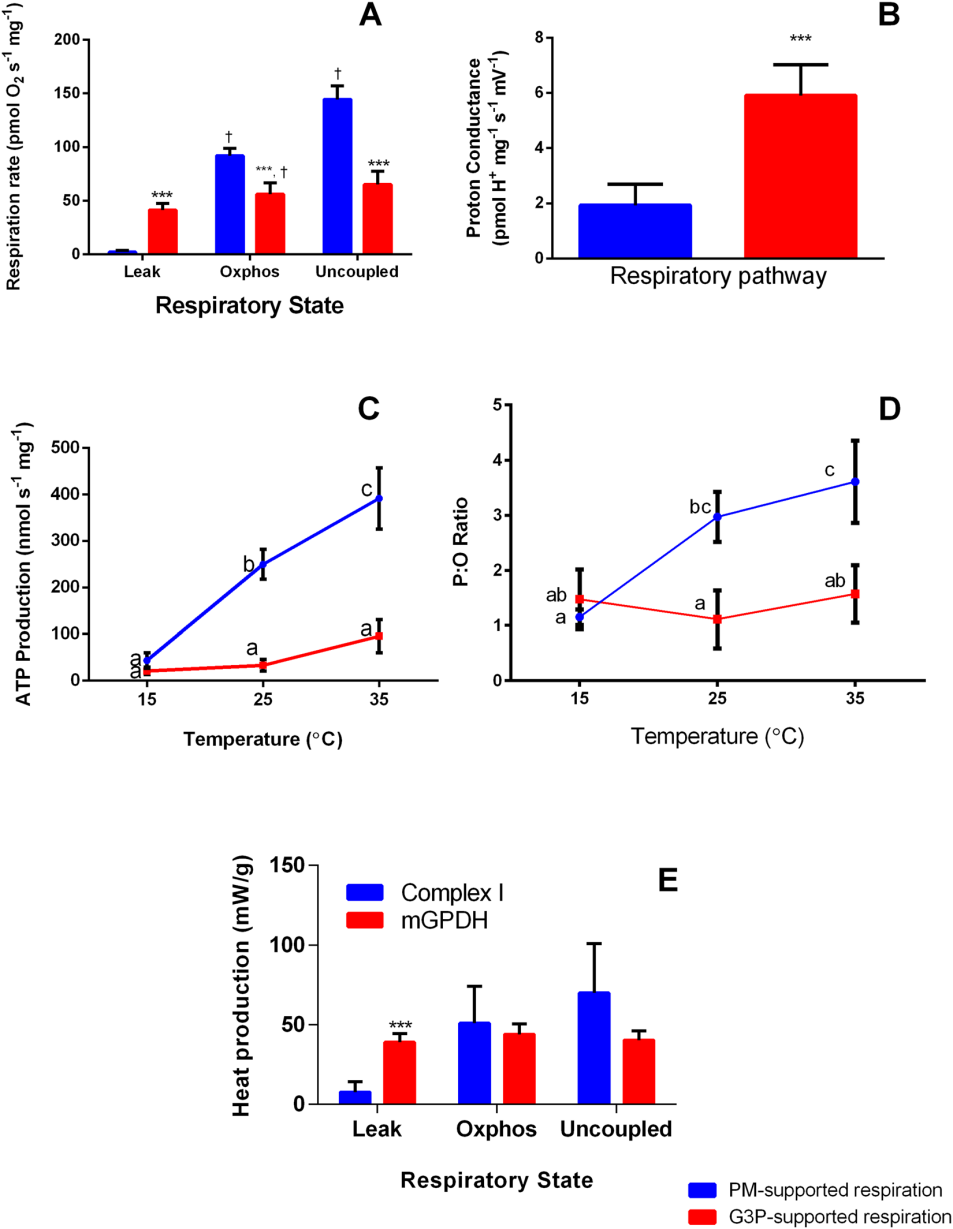
Comparison of pyruvate-malate and G3P-supported oxidation efficiency. (**A**) Respiration rates at 15 °C supported by either pyruvate and malate (PM) (n = 6) or glycerol 3-phophate (G3P) (n = 5) in *B. terrestris* flight muscle mitochondria. (**B)** Inner membrane proton conductance under PM supported (n = 5) or G3P (n = 5) supported respiration. (**C)** PM (n = 5) and G3P (n = 4) supported ATP Production Rates at 15, 25, and 35 °C (**D**) P:O ratio of PM and G3P supported respiration at 15, 25, and 35 °C. (**E)** Heat production at 20 °C from PM (n = 5) and G3P (n = 6) supported respiration Data are mean ± s.e.m of at least four replicates. Letters (a, b, c, d) denote significant differences from other states and respiratory pathways (p < 0.05). ***P < 0.01 between respiratory pathways, ^†^ *P* < 0.01 between respiratory states, two-sample Student’s t-tests for pair-wise comparisons, linear mixed models for multiple comparisons.

Oxygen flux in the presence of high ETS substrate, and the absence of ADP (leak respiration) of permeabilised muscle fibres was 18-fold greater during G3P-supported respiration than during pyruvate + malate supported respiration (PM) at 15 °C (Fig. 1A). High G3P-supported Leak respiration has been previously described for this species^13^, but never in the context of thermogenesis. PM-supported leak respiration was 2.5-fold more sensitive to temperature (temperature coefficient, Q_10_, of 3.6) than G3P-supported respiration (Q_10_ of 1.42), this means G3P-supported leak respiration can achieve high flux at lower temperatures.

Due to disparity between PM and G3P-supported leak, we determined inner-membrane proton conductance through simultaneous measurements of oxygen flux and membrane potential^20,21^. Proton conductance was 3-fold higher during G3P-supported leak respiration than during PM-supported leak respiration (Fig. 1B). This suggests mGPDH activity elevates proton conductance of the inner membrane, a phenomenon observed in yeast^22^. As mGPDH is the greatest contributor to ROS production in *B. terrestris* flight muscle^23^, and ROS have signalling roles^24,25^, mGPDH-derived ROS may facilitate the observed increase in proton conductance through redox signalling, or modification of existing membrane constituents.

Measurements of ATP production in the cold showed ATP synthesis is negligible from either G3P or PM-supported respiration at 15 °C (Fig. 1C). As shivering and futile cycling mechanisms rely on ATP, the lack of ATP synthesis from either G3P or PM-supported respiration at 15 °C will likely suppress the heating capacities from these mechanisms. In addition, for PM-supported respiration the dynamic P:O ratios (an index of the amount of ATP produced per oxygen molecule consumed) increased with temperature (1.15, 2.96, and 3.60 at 15, 25 and 35 °C respectively), while for G3P-supported respiration the dynamic P:O ratios remained low at all temperatures (1.47, 1.11 and 1.57 at 15, 25 and 35 °C, Fig. 1D). This indicates that PM-supported OXPHOS becomes progressively more efficient at producing ATP as temperature increases, while G3P-supported OXPHOS remains inefficient across temperatures.

To resolve whether sufficient heat production coincides with high oxygen flux we built a calorimeter that permitted mixing and gassing, and also multiple substrate titrations onto permeabilised flight muscle preparations. In accordance with the respiration rates, G3P-supported leak respiration produced 5-fold more heat than PM-supported (Fig. 1E). Given the heat capacity of muscle is approximately 3.421 J g^−1^ °C^−1^ ^26^ and bumblebee flight muscle releases 36 mW g^−1^ (36 W kg^−1^, 38.6 × 10^3^ W m^−3^) of heat solely through G3P-supported leak respiration, we estimate it would take 37 minutes to warm an average (55 mg) bumblebee thorax from 15 °C to 25 °C. Reported rates of thermogenesis suggest *B. terrestris* can warm from 6 to 36 °C in 15 minutes^3^. While this is much faster than the 37 minutes that we estimate, our calculations do not incorporate dynamics of heating, where mGPDH activity will increase with temperature, and therefore increase the heating rate. In addition, PM-supported respiration will increase as temperatures rise, contribute to thermogenesis, and support OXPHOS. The latter will also provide ATP to power shivering and/or futile cycling. Taken in context, the rate of heating achieved via mGPDH-mediated flux could supplement other thermogenic mechanisms such as shivering in warming flight muscles, to pre-flight temperatures.

**Figure 2:**
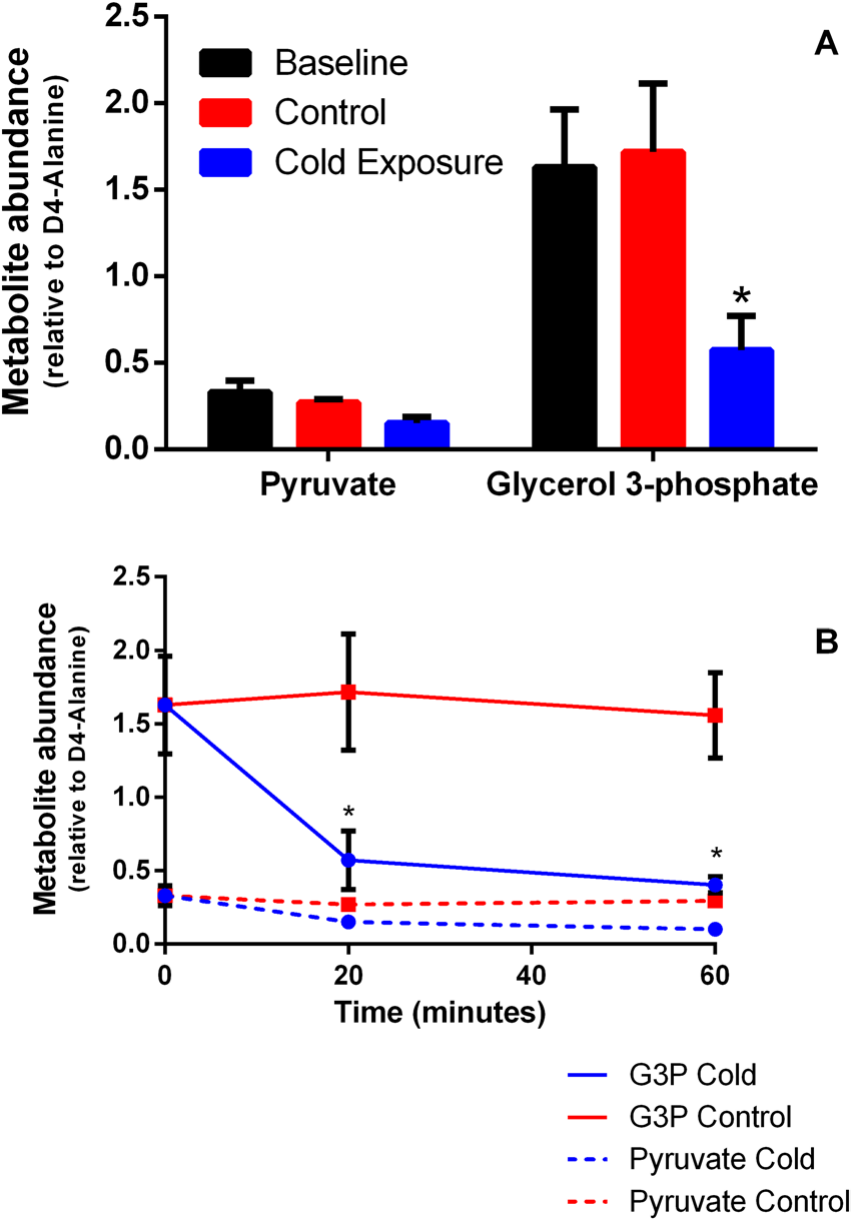
Effect of cold exposure on substrate usage by *B. terrestris* flight muscle. (**A**) Relative (to internal standard, D4 Alanine) abundance of pyruvate and G3P (n = 5) directly after capture (active), and after 20 minutes of either 25 °C exposure (rested normothermic) or 4 °C (cold exposure). (**B**) Relative abundance of pyruvate and G3P at 20 and 60 minutes, comparisons made between cold (4 °C) and rested normothermic (25 °C) groups. Data are mean ± s.e.m. *P < 0.05, two-sample Student’s t-tests for pair-wise comparisons, two-way analysis of variance for multiple comparisons.

If G3P-supported respiration promotes mitochondrial heat production, and PM-supported respiration is impaired in the cold, G3P concentrations within flight muscles should decrease following cold exposure, while pyruvate concentration should either increase or remain unchanged. Using gas-chromatography mass-spectrometry, we compared muscle metabolites from bumblebees chilled to 4 °C against rested bumblebees held at 25 °C, and active bumblebees frozen immediately after capture. After 20 minutes of cold exposure, G3P content decreased 3.2-fold (Fig. 2A) relative to active and rested animals, while pyruvate content remained similar among groups. This indicates that bumblebee flight muscles deplete G3P pools at low temperature. After 60 minutes, this relationship was maintained (Fig. 2B). We contend that changes in G3P relative to pyruvate, reflect the maintenance of G3P oxidation by mGPDH and the cold-inhibition of PM-supported respiration.

It is possible that the observed changes in G3P relative abundance result from a cold-mediated decrease in glucose metabolism, however we observed no differences in glucose, G6P or pyruvate concentrations (Supplementary Information, Fig. 1). This indicates that there is no inhibition of pyruvate production as the end product of glycolysis, nor is there a concomitant increase in glucose concentration indicating slowed breakdown of glucose. Thus, the difference in G3P concentration is not explained by a lack of glucose breakdown, and occurs without resulting limitation of pyruvate production. It is possible that other anapleurotic reactions contribute glycolytic intermediates, but in the context of the respiration and calorimetry data, we believe it is fitting to suggest that G3P is preferentially oxidised to produce heat.

Taken together our results show that *B. terrestris* may warm flight muscles using the mGPDH respiratory pathway. Moreover, PM-supported respiration through CI is impaired in cold flight muscles, and G3P-supported respiration through mGPDH has much lower temperature sensitivity. Notably, the poor ATP synthesis capacity of PM-supported OXPHOS at low temperatures will depress ATP synthesis required for futile cycles or shivering. Overall, the poor phosphorylation efficiency of mGPDH can support adequate heat production to pre-warm cold bumblebees, and presents a thermogenic role for mGPDH.

## Methods

### Animal procedures and ethics statement

Animals were bought from a commercial vendor (Biobees Limited, Hastings, New Zealand) and stored on campus in a flower garden with access to a range of flowers and protection from inclement weather. All procedures met the ethics guidelines of the University of Auckland.

### Tissue preparation

Animals were cold anaesthetised (4 °C) for 20 minutes before decapitation. All dissections and permeabilisation process were conducted on ice at 4 °C, and all chemicals were purchased from Sigma-Aldrich unless otherwise specified. Flight muscles were rapidly excised and placed in ice cold preservation solution (10 mM Ca-EGTA buffer, 0.1 µM free calcium, 20 mM imidazole, 20 mM taurine, 50 mM K-MES, 0.5 mM DTT, 6.56 mM MgCl_2_, 5.77 mM ATP, pH 7.1 at 0 °C). Tissue permeabilisation followed an adapted, previously described protocol^27^. Muscle fibres were exposed to 5 mg ml^−1^ saponin for 30 mins before being washed 3 times for 10 mins with mitochondrial respiration media (MiR05; 0.5 mM EGTA, 3 mM MgCl_2_∙6H_2_O, 60 mM lactobionic acid, 20 mM taurine, 10 mM KH_2_PO_4_, 20 mM HEPES, 110 mM D-Sucrose, 1 mg ml^−1^ fatty acid free BSA). All chemicals were purchased from Sigma-Aldrich unless otherwise specified.

### High resolution respirometry

Respiration protocols were conducted using O2k Oxygraphs (Oroboros Instruments, Innsbruck, Austria). Media oxygen concentration was maintained between 500 and 200 nmol ml^−1^. Between 1–2 mg of tissue was added to each 2 ml chamber. PM-supported respiration assays contained 5 mM pyruvate and 2 mM malate, while G3P-supported respiration assays contained 10 mM G3P. After addition of ETS substrates ADP was added in saturating concentrations (previously experimentally determined as 1 mM for mGPDH assays and 5 mM for Complex I assays). The uncoupler CCCP was then titrated in 0.5 μM steps until respiration rate ceased to increase.

### Determination of Inner Membrane Proton Conductance

Proton conductance was determined based on work by Brand^20,21^, under the assumption that in the absence of ADP, the ATP_Fo-F1_ is not functional, and any oxygen flux can be attributed to the maintenance of ∆Ψm against proton leak. First, mitochondrial membrane potential MMP was estimated simultaneously with respirometry at 20 °C using a modified protocol from Krumschnabel *et al*.^28^, which has also been used on insects such as *D. melanogaster* ^29^. Safranin fluorescence (ex/em, 495 nm/587 nm) was calibrated by a four-step titration to a final concentration of 2 µM. MMP was calculated from the recorded calibrated concentration of safranin according to the Nernst equation, with an assumed membrane potential of 180 mV during OXPHOS. While this is the value taken from mammalian mitochondria^30^, prior work on *B. terrestris* has suggested high similarity to mammals with respect to MMP^13^. Based on known substrate dependent proton fluxes (20 and 12 per oxygen for NADH and FADH_2_ linked substrates respectively, i.e. PM and G3P respectively), respiration rate was converted to proton flux. Proton flux was then standardised against membrane potential to account for the non-ohmic relationship between increasing membrane potential and proton leak^31^.

### Measurement of ATP production

ATP production was measured simultaneously with respiration rates using Magnesium Green™ (MgG, ThermoFisher Scientific, Waltham, MA, USA) fluorescence (ex/em, 503 nm/530 nm), based on previous work^32–34^. Flight muscle was prepared as previously described before being added to the chamber. MgG™ (5 µM) was added to 2 ml of respiration media in the presence of 30 µM ouabain to inhibit the Na^+^/K^+^ -ATPase, 50 µM Adenosine-5 pentaphosphate for adenylate kinase inhibition, 0.05 µM blebbistatin (Cayman Chemical Company, Ann Arbor, MI, USA) to inhibit myosin ATPase and 1.25 μM thapsigargin (Abcam, Melbourne, VIC, Australia) to inhibit the smooth endoplasmic reticulum calcium ATPase. PM and G3P-supported respiration protocols were conducted as described above. ATP production signal was calibrated based on previous work by conducting a separate titration of ATP and ADP up to 5 mM^32^. ATP production rates were then determined by expressing this change in ATP concentration over time in Excel (Microsoft, Redmond, WA, USA).

### Calorimetry

An in-house, purpose built, calorimeter (see Supplementary Methods) was used to measure heat production from permeabilised muscle fibres. The above tissue preparation and respiration protocol was followed, instead following heat production. Samples were measured against a reference chamber which received all media and substrates without tissue samples.

### Measurement of muscle metabolites following cold exposure

Bumblebees were held at 4 °C for 0, 20, or 60 minutes, before being snap-frozen in liquid nitrogen. At the two cold-exposure time points, a control bee left at 25 °C was also snap-frozen to account for changes in metabolite concentration over time, independent of cold exposure. The head, abdomen, wings and legs of each bee were removed. The thorax was weighed and stored at −80 °C for future analysis.

Gas chromatography-Mass spectrometry methods were based on previous work^35–41^. To extract metabolites, whole thoraxes were freeze dried overnight at 0.084 Torr and −84 °C before being homogenised and weighed. Samples underwent a two stage extraction, first in 50% MeOH:H_2_O, and then again in 80% MeOH:H_2_O, at this point the internal standard, D4-alanine was added. The supernatants from these two extractions were combined and concentrated using a SpeedVac (Savant SC250EXP SpeedVac Concentrator and Savant RV5105 Refrigerated Vapour Trap, Thermofisher Scientific, Waltham, MA, USA). Samples were desiccated overnight, and then resuspended in 80 μL of 2% (w.v.) methoxyamine hydrochloride in pyridine. The contents of each tube were transferred to a GC vial containing a silanised insert, and incubated at 30 °C for 90 minutes. Samples were removed and 80 µL of MSTFA (N-methyl- N[trimethylsily]trifluoroacetamide, Merck, Darmstadt, Germany) added, before returning to the oven for 30 minutes at 37 °C.

One microliter of sample was injected into an Agilent gas chromatograph at with the use of a CTC PAL auto-sampler. The column was a ZB-1701, and carrier gas was instrument grade helium (99.99%, BOC). The temperature of the GC was initiated at 70 °C for 5 min, and then increased 10 °C min^−1^ to 179 °C for 5 min. Total run time of samples in the GC machine was 41.286 min. Following the GC process, samples were transferred automatically via a transfer line to mass spectrometric detector which was maintained at 250 °C, the MS source at 230 °C and MS quadrupole at 150 °C. Dichloromethane blank was run before and after samples to monitor instrument carryover. The identification of compounds was determined based on scanning of the mass spectra from 15 to 500 atomic mass units and abundances were presented relative to the internal standard, D4-Alanine.

### Statistics

No statistical tests were used to calculate appropriate sample sizes. Treatments were randomised between machines and individual animals where appropriate. Respiration rate, ATP production, and calorimetry data were analysed in R platform using linear mixed effect models to assess differences between respiratory states, pathways, and/or temperatures. Post-hoc, pairwise comparison adjusted, two-sample t-tests were used to determine differences in means. Residual plots indicated respiration rates from the ATP assays, and calorimetry data violated assumptions of equality of variance, therefore these data were log-transformed before analysis. Animals were randomly allocated to cold exposure groups. Mass spectrometry analyses were blinded to treatment group. Metabolite levels were compared using GraphPad Prism 6 (GraphPad, La Jolla, CA, USA). Main effects were compared via two-way ANOVA while pairwise comparisons were made using Tukey’s post-hoc test. Membrane conductance was also compared in Prism 6 via an unpaired t-test.

## Electronic supplementary material

Supplementary Information
