# Supplementary material for: Mitochondrial glycerol 3-phosphate facilitates bumblebee pre-flight thermogenesis
Source: Sci Rep. 2017 Oct 12;7:13107. doi: 10.1038/s41598-017-13454-5 (PMC5638826; doi:10.1038/s41598-017-13454-5)
Supplement: Supplementary file 1 — Supplementary Information [file 41598_2017_13454_MOESM1_ESM.pdf]

Title: Mitochondrial glycerol 3-phosphate facilitates bumblebee pre-flight thermogenesis

Authors: Stewart W.C. Masson[1], Christopher P. Hedges[1, 2], Jules B. L. Devaux[1], Crystal S. James[1] and Anthony J. R. Hickey[1]*

Supplmentary Results

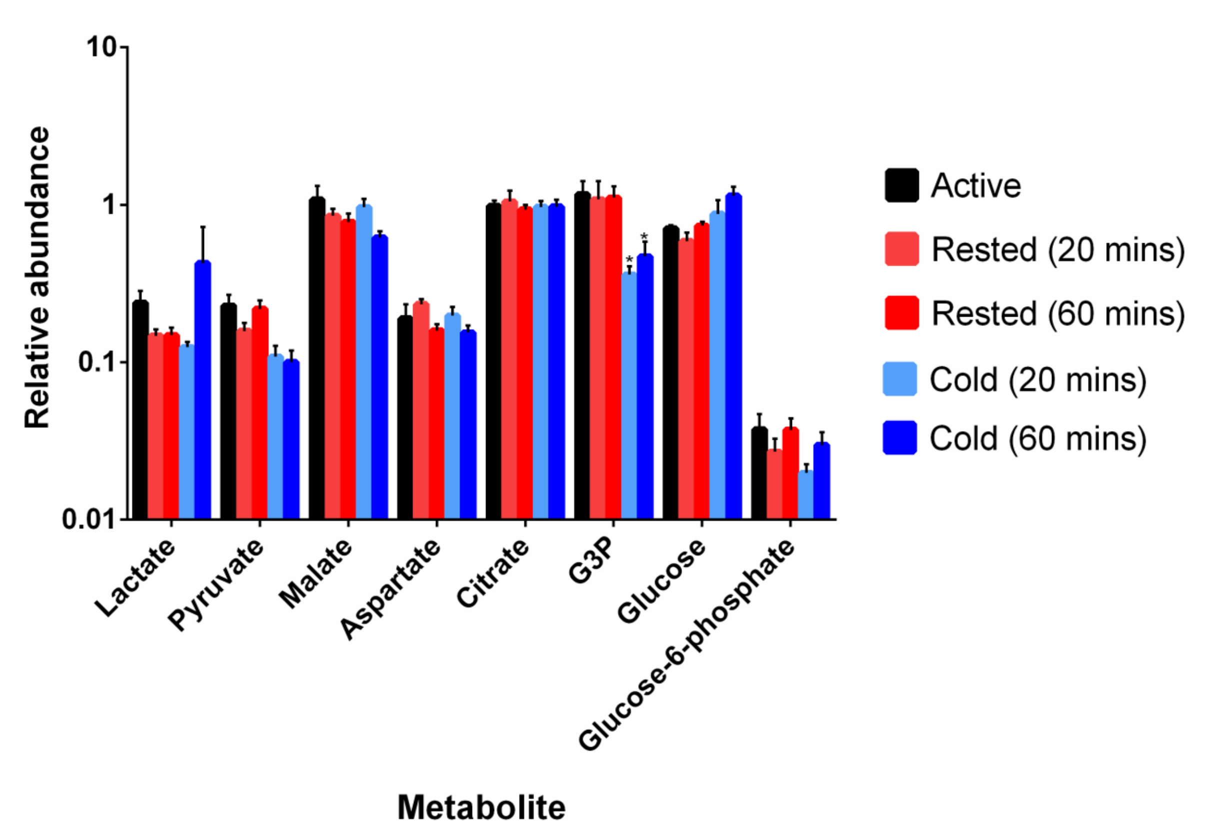

Supplementary Information Figure 1 Relative abundance of various glycolytic and citric acid cycle intermediates across control, rested and cold exposed groups, data is presented on a log axis to make visualisation of each metabolite easier. Data are mean ± s.e.m of 5 replicates. **P* <0.05, two-sample students t-tests for pair-wise comparisons, two-way analysis of variance for multiple comparison.

Supplementary Methods

Six TEC-12706 Peltier pads were attached to a Gold-anodised, aluminium heat sink with thermal grease. Aluminium wells, measuring 10mm in height, 38mm in diameter for a total volume of 11ml were attached to the top of the Peltier pads with more thermal grease. The heat sink and wells were all insulated and isolated from each other with polystyrene. Three of the wells functioned as control chambers while the other three received samples. The heat-sink, Peltier pads, wells were all enclosed in polystyrene which was then placed inside a larger polystyrene box producing a double layer design. This was then placed inside a 20°C temperature controlled room. This larger box was fastened to a Stuart SSM1 mini-shaker table (Bobby Scientific Limited, Staffordshire, United Kingdom) which allowed mixing of the wells without interference by a stirrer rod or bar. Each Peltier pad was connected via shielded coaxial cable and attached to an SP series 16/s AD Instruments Power lab (AD Instruments, Dunedin, New Zealand). Data was collected using Lab Chart 7 (AD Instruments). The signal from the control chambers was subtracted from the experimental signal using Lab Chart. The calorimeter was calibrated by filling each chamber with respiratory media and submerging a resistor which produced known wattages. This value was then compared to the voltages detected by the calorimeter, observed as a mV response in Lab Chart (Supplementary Methods, Figure 1). Substrates and inhibitors were injected into the chamber via tubing attached to syringes outside the calorimeter. This prevents heat being introduced to the chamber by the user during injection of compounds. Data generated by our calorimeter was validated by comparing against data generated in other studies using similar in-house designs (Supplementary Methods, Table 1). It was found to be within the range of previously generated values from other highly-energetic tissues.

Heat Production Value (mW/g)	Organism and Tissue	Physiological Parameter	Experimental Temperature	Reference
**39.2 ± 5.4**	***Permeablised B. terrestris* flight muscle**	**Leak respiration with G3P as a substrate**	**20°C**	**–**
22.2 ± 1.6	Cat cardiac muscle	Contracting muscle with caffiene and Ca^{2+}	25°C	36
37.6 ± 5.2	Guinea pig cardiac muscle	Contracting muscle with caffiene and Ca^{2+}	25°C	36
30 ± 2.8	Rat cardiac muscle	Contracting muscle with caffiene and Ca^{2+}	25°C	36
0.53 to 0.75 (inter quartile range)	Human vastus lateralis	"Resting metabolism" with glucose	37°C	37
12 to 17 (range)	Tent moth flight muscle	Shivering	20°C	38
18.3 ± 1.5	Permeablised guinea pig cardiac muscle	Leak respiration with PM as substrates	37°C	39
36 ± 8	Permeablised guinea pig cardiac muscle	Leak respiration with glucose and pyruvate	37°C	39
"Up to 170" (reported value)	Permeablised guinea pig cardiac muscle	Uncoupled respiration with glucose and pyruvate	37°C	40
106 ± 40 (dry weight)	Permeablised guinea pig cardiac muscle.	Leak respiration with PM as substrates	37°C	41

Supplementary Methods Table 1 Table of experimentally determined calorimetry values used to validate our calorimeter with references.

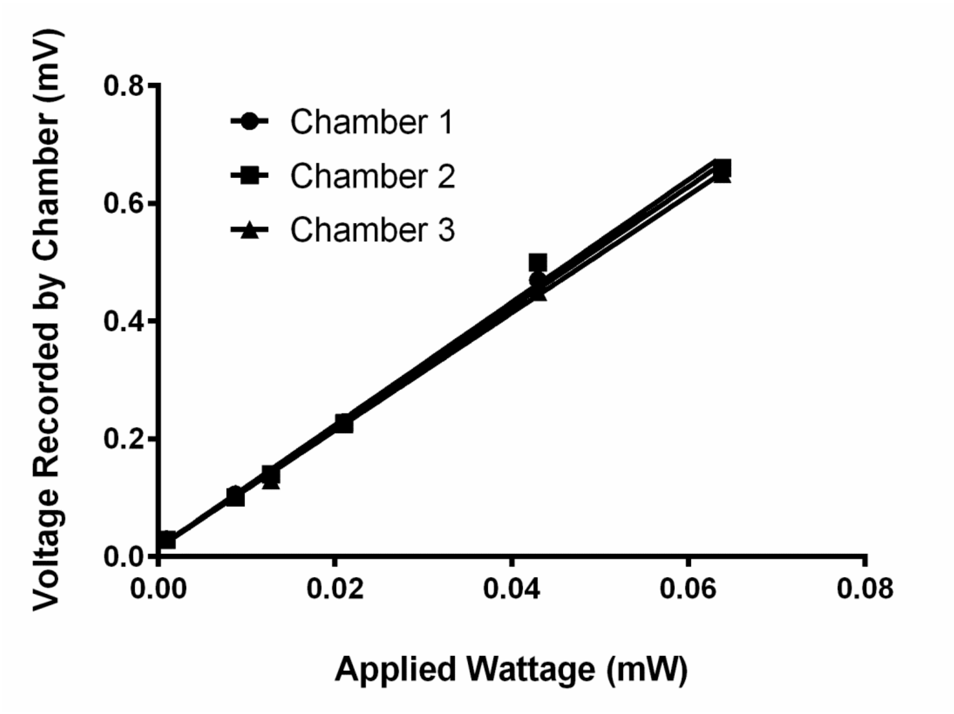

Supplementary Methods Figure 1 Calibration curve of purpose built calorimeter.
